# A Conceptual Framework for Multi-Dimensional Measurements of Food Related Pleasure—The Food Pleasure Scale

**DOI:** 10.3390/foods10092044

**Published:** 2021-08-31

**Authors:** Barbara Vad Andersen, Raymond C. K. Chan, Derek Victor Byrne

**Affiliations:** 1 Food Quality Perception and Society, Department of Food Science, Aarhus University, Agro Food Park 48, DK 8200 Aarhus, Denmark; derekv.byrne@food.au.dk; 2 Sino-Danish Center for Education and Research, Beijing 100190, China; rckchan@psych.ac.cn; 3 Neuropsychology and Applied Cognitive Neuroscience Laboratory, CAS Key Laboratory of Mental Health, Institute of Psychology, Chinese Academy of Sciences, Beijing 100049, China

**Keywords:** food, pleasure, framework, individual differences, scale development

## Abstract

In modern times, the majority of food intake is believed to be driven by hedonic processes, rather than homeostatic ones. Various factors have been found to influence the hedonic eating experience and thereby influence eating behaviour, and each factor can be regarded a piece that contributes to parts of the total picture of the hedonic response to food. As a result, the literature on the hedonic response to food-related experiences is comprehensive, but at the same time rather fragmented; and importantly, it is not clear how individuals/segments differ in key drivers of their hedonic experience and the extent to which food pleasure is perceived. In this paper, we present a conceptual framework for the development of a scale (self-report questionnaire) to measure the qualitative and quantitative aspects of food-related pleasure, the Food Pleasure Scale. We introduce the concept of (an)hedonia and scales developed in the past for its measurement, identify the spectrum of characteristics influencing food-related pleasure and explain the relevance of developing such a scale. Based on this theoretical framework, a strategy for the development of the Food Pleasure Scale is proposed.

## 1. Introduction

Hunger and satiety sensations, and the energy balance, are regulated by a neuroendocrine system located in the brain—in the hypothalamus. This system involves a network of neurohormonal circuits responding to signals of peripheral and central origin; the homeostatic system; and factors of sensory, mechanical and cognitive origin—the hedonic system. The hedonic system is associated with the activation of the neuronal reward system, which produces a pleasurable sensation. Resent research shows that human eating behaviour is largely driven by the pleasure generated from engaging in food-related experiences, both the healthy regulation of intake and intake that may cause overconsumption or underconsumption [1,2,3]. If eating behaviour were only regulated by homeostatic systems, food intake would be driven by a response to a purely physiological need, and as a result, the majority of people would be of normal weight. However, human appetite is more complex than that, as we experience pleasure when perceiving the food or even just thinking about the food; and the context the food is presented in does not matter, regardless of a physical or social setting. All of these factors (and more) thereby have the potential to influence human foraging and consummatory behaviour. The study of pleasure from food-related experiences has been approached from many different perspectives, including neuroscience, health-and consumer science and even various perspectives within the same discipline, as illustrated in this paper focusing on sensory and consumer science (see Section 5, Table 1), resulting in a comprehensive but also fragmented picture of the various different factors influencing the subjective pleasurable eating experience. Therefore, it is not clear what the key drivers of (dimensions underpinning) the subjective pleasurable eating experience are, and how individuals differ in what drives their pleasure and the importance of each driver. While each of the previous perspectives has shed light on parts of the total hedonic picture, analysing the drivers in an integrated manner will provide a more holistic picture and detail the complexity of the subjective hedonic eating experience.

**Figure 1:**
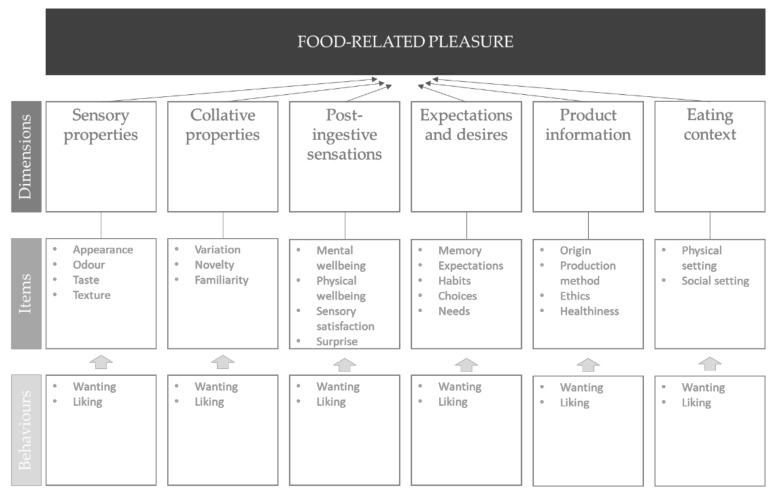
A schematic conceptualisation of the key dimensions, items, and behavioural elements involved in the individual food-related pleasure response, allowing a holistic study of quantitative (level of pleasure) and qualitative (drivers of pleasure) aspects of food-related pleasure.

In this paper, we bring together, via the literature, the elements that are necessary for the holistic study of quantitative (level of pleasure) and qualitative (drivers of pleasure) aspects of food-related pleasure (see Section 6, Figure 1). We focus on individual difference characteristics that have the possibility to form a general pattern across populations sharing characteristics (instead of specific eating situations). This is important, as the stability of the hedonia construct and/or its sub-dimensions is uncertain. It is well-know that individuals with somewhat unstable personality traits, such as neophobia, have peculiar food-related behaviour [4]. At the same time, it is well-known that the ability to perceive pleasure can be impaired when suffering from, e.g., depressive disorders and schizophrenia [5,6,7,8]. Together, these trends show alterations in the otherwise relatively stable construct of pleasure, depending on the health condition of the subject. It would be desirable to be able to test the stability of the pleasure experience and the underlying drivers. Thus, we define food pleasure as a state we, as humans, strive towards and that is underpinned by several drivers. The relative importance of each driver to the level of pleasure experienced can differ between individuals, and can be altered by health-related circumstances affecting the biological reward system. We believe it is necessary to study the individual drivers and their relative importance levels for subjective levels of food pleasure in an integrated manor, in order to understand the pleasurable eating experience and the ways by which it can manifest in certain eating behaviours.

Specifically, we introduce the concept of (an)hedonia, the (in)ability to experience pleasure, and scales developed in the past for its measurement (Section 2), and unfold the relevance of approaching (an)heonia in a food context (Section 3). Further, we identify key components influencing food-related pleasure to show the complexity and relevance of this research field (Section 4 and Section 5), and with this background, we introduce a theoretical framework for the development of a food pleasure scale (Section 6).

The theoretical framework presented here will be used to develop a self-reported scale to measure qualitative and quantitative aspects of food pleasure (outside the scope of this paper). Such a scale will facilitate researchers to accurately tap into the subjective nature of what individuals find pleasurable in food-related contexts; identify subjects with altered or impaired hedonic response(s) and the characteristics of these populations; and clarify the underlying causes. Greater insights into the cognitive processing of food pleasure cues will be important for understanding the unique flexibility of human food choices and the conditions that might promote eating behaviours (including overeating), and be relevant for guiding people toward healthy eating behaviours.

## 2. Understanding Food Pleasure in Relation to the Generally Used Definition of (an)Hedonia

Traditionally, anhedonia has been defined as the inability to experience pleasure [9]. The definition refers to both a symptom in various psychiatric disorders and a personality trait, and emphasises the “consummatory” aspect of the reward function. Over the years of research toward developing a scale to measure anhedonia, a broader conceptualisation has been used which besides “consummatory pleasure” also includes “interests” in response to stimuli that are perceived as rewarding [10]. Consequently, it was suggested that it may be more useful to explicitly understand and use anhedonia as an umbrella term for impairment of hedonic function over a spectrum of behaviours reflecting “anticipatory” and “consummatory” pleasure [11].

Four main validated self-report measures have traditionally been used in clinical research to assess (an)hedonia: the Snaith–Hamilton Pleasure Scale (SHAPS) [12], the Fawcett–Clark Pleasure Capacity Scale (FCPS) [13], the Revised Chapman Physical Anhedonia Scale (CPAS) and the Chapman Social Anhedonia Scale (CSAS) [14]. Later, the Temporal Experience of Pleasure Scale (TEPS) was developed [15], and the Dimensional Anhedonia Rating scale (DARS) was developed and validated [11]. Ideally, a scale quantifying anhedonia should be able to detect temporary conditions (state) from long-term characteristics (trait), be able to measure different facets of anhedonia and be appropriate for use in samples with different cultural backgrounds and individual preferences (generalisability). These four measures differ in their ability to address these factors (see [11] for a detailed comparison of the scales).

As can be seen in the development of scales to measure pleasure, the clinical setting has dominated for measurement of anhedonia. Though the before-mentioned requirements should be seen primarily from a clinical perspective, they can be generalised as requirements for a scale aimed at broader or different applications, such as defining the pleasure associated with food-related experiences. The clinical scales commonly address the following domains (with few exceptions or small variations): social interaction, interest/pastimes, sensory experience and food/drink. However, the equivocal conceptualisation of these scales makes anhedonia measurements difficult and imprecise. This is problematic for symptom definition, understanding the biological underpinnings of anhedonia and defining what anhedonia is presently understood to encompass. In our conceptualisation of pleasure, the focus is on pleasure derived from food-related behaviours. As such, the term is made univocal by focusing on one of the domains commonly used in anhedonia scales (namely, food), though we acknowledge that even with this more precise view, the domain of food pleasure should be understood as an umbrella term for hedonic function over a spectrum of food-related behaviours. The specific behaviours underneath “the food hedonia umbrella” still need to be brought together from the comprehensive scientific literature to present a total holistic picture and be validated, but later in this paper we present a picture of its complexity (Section 5, Table 1).

## 3. The Relevance of Addressing (an)Hedonia in a Food Context

For the vast majority of human history, the primary reason for seeking food was linked to survival by maintaining energy homeostasis and avoiding starvation. As such, homeostatic hunger is related to restrictions in energy intake over long periods of time. In modern times, among well-nourished populations, most food consumption occurs for reasons other than acute energy deficiency. The term “obesogenic environment” has entered the scientific vocabulary, and implies that the external environment is partly responsible for the increases in food intake and is one of the underlying causes of intake beyond homeostasis and obesity. This approach has led to an interest in the sensory and external factors influencing food intake and has increased scientific awareness of the hedonic influence on appetite. There is now a strong belief that a major cause of the increased food intake associated with obesity is related to the hedonic system rather than the homeostatic system [1]. Historically, hedonic processes were normally caused by a nutritional need-state. In a state of depletion, the rewarding effects (experienced pleasure) of energy-providing foods are enhanced, and when replete, the rewarding effect of these foods is reduced [16]. This view supports a link between energy density and palatability [17], and posits that neurological processes control the consumption of energy-dense nutrients [18]. However, the idea of reward as a consequence of the fulfilment of nutritional needs does not explain food intake beyond homeostatic needs. Therefore, hunger may arise more commonly from anticipated pleasure of eating, while being subject to feedback control from homeostatic and cognitive processes.

To clarify how the two terms “homeostatic” and “hedonic” hunger can be distinguished, some examples follow here. The chronic hunger a dieter might experience while losing weight is homeostatic in nature, so is the eating driven by fasting. On the other hand, hedonic hunger can be exemplified as the desire for sweets a weight-stable person would experience after a filling meal. A sudden desire for a bun in the middle of the afternoon after walking by a bakery and smelling fresh buns being made is another example. These examples provide a sense of why hedonic hunger can become a powerful influence on food intake, and ultimately, body mass.

Most non-homeostatic mechanisms are related to the brain’s reward system. Understanding their role is a research priority. Until recently, most studies have focused on the role of homeostatic signals in appetite regulation, such as metabolic hormones and the availability of nutrients in the blood. However, interest in understanding how humans eat in a nonregulated manner, or beyond metabolic needs, has become a priority in recent years [19].

Several pleasure dimensions can have separate roles in overconsumption. In terms of enjoyment (consummatory pleasure), some individuals may experience augmented hedonic responses to palatable foods, and food can then be eaten in greater amounts because of being enjoyed more [2,3]. Conversely, some individuals may experience less pleasure from food, and therefore, consumption of food may be driven up to satisfy an optimum level of stimulation [20]. Wanting-related processes (anticipatory pleasure) may also lead to weight gain through increased reactions to cues from available foods [21], and a reduced ability to resist further eating despite being satiated [22]. It can be hypothesised that wanting may be the central process in prompting and maintaining obesity. For example, research on chronic drug abusers shows that the subjects process high levels of wanting (motivation) for a “fix,” despite not felling pleasant sensations during ingestion [23]. 

Taken as a whole, the scientific literature suggests that the hedonic and rewarding effects of foods are multiple and highly involved in human eating behaviour, but insight into the individual hedonic processes is broadly lacking. A scale measuring food-related pleasure will, as the first step in a sequence of studies, facilitate: accurately tapping into the subjective nature of what individuals find pleasurable in food-related contexts; identifying subjects with different or impaired hedonic response(s) and the characteristics of these populations; and clarifying the underlying causes. Greater insights into the processes of food pleasure cues will be important for understanding the unique flexibility of human food choices and the conditions that might promote overeating, and will be critical for understanding and guiding people toward healthy eating behaviour.

## 4. The Application Potential of a Pleasure Scale within the Food Domain

In this section, relevant areas of usage within the food domain are described. The section focuses on the relevance of the scale for understanding drivers of overconsumption. However, other more applicable-oriented usage options include: clinical purposes, where the scale could be used for the diagnosis of eating disorders and to understand the food-related pleasurable alterations; and strategic marketing, where the underlying drivers of pleasure could be used to create profiles of consumer segments, and market food concepts based on factors of major importance for each segment’s pleasure experience.

Global estimates of the prevalence of overweight and obesity demonstrate that more than 1.9 billion adults are overweight, and of those, 600 million are obese [24]. Said diseases greatly elevate the likelihood of non-communicable diseases, ill health and shortened life spans. The growing prevalence of global obesity suggests that an increasing proportion of human food consumption is not just driven by the need for calories but by pleasure. This “positive-incentive” perspective suggests that high calorie foods have had intrinsic reward value throughout evolution, meaning that the need for energy and the benefit of high caloric foods makes people crave them [25,26]. Thereby, the presence of desirable (hedonic) food, or the mere anticipation of it, makes one hungry [27]. The psychological effects of hedonic hunger may even be the appetitive equivalent of hedonically-driven activities such as recreational drug use and compulsive gambling. Such hedonistic eating overrides the body’s ability to regulate consumption with satiety [28]. Hedonic appetite preference may lead to increased weight gain due to eating when not hungry and in need of energy. The availability and palatability of foods have major effects on wanting for these foods and subsequent food intake, no matter of the need-state of the individual.

From a scientific perspective, key questions include: do people who gain weight and become obese have different pleasure responses from people who remain lean? To answer this and related questions, a scale to measure quantitative and qualitative aspects of food pleasure is of outermost relevance. Additionally, if the answer to this question is “yes,” do people suffering from obesity then experience higher or lower hedonic appetites than lean people? Put another way, does obesity relate to a suppressed or a super-sensitive pleasure response to food? These questions are theoretically important, since both possibilities could account for over-eating. If food intake is linked to a low pleasure response, it could be argued that people would need to eat more food in order to gain an adequate level of pleasure (anhedonia manifests as additional eating). Conversely, if food results in a high pleasure response, this could stimulate more eating in order to gain maximal pleasure. A scale measuring the pleasurable response(s) will allow us to quantify the hedonic food experiences and determine differences between individuals/populations, and allow us to measure different aspects of pleasure and detect state versus trait differences.

## 5. Key Components Influencing Food Pleasure

A broad range of factors have been stated in the scientific literature to influence the pleasurable eating experience. With the aim of illustrating the comprehensive yet fragmented number of studies focusing on food-related pleasure and clarify relevant dimensions for a scale measuring food-related pleasure, we present, in this section, a selection of key food and eating-related behavioural factors influencing food pleasure (overview presented in Table 1).

### 5.1. Sensory Characteristics

Firstly, the intrinsic product characteristics, such as the sensory properties, including the food’s appearance, odour, taste (flavour) and texture. The importance of sensory properties for a pleasurable eating experience is supported by a broad range of studies and models focusing on: sensory properties and acceptance—e.g., [29,30,31]; sensory properties and preferences—e.g., [32]; sensory properties and food behaviour—e.g., [33,34,35,36]; and sensory properties and food satisfaction [37,38,39]. Together, these show how sensory properties evoke a hedonic component in addition to the sensory perception, which can influence one’s motivation to eat, thereby facilitating continued eating and additional desires; and how sensory properties are among the primary drivers of food-related pleasure (here satisfaction). Scales applied in clinical research to assess anhedonia also focus on the sensory food experience, by addressing taste [14,15] or by referring to favourite foods [11,12,15], which points at the outermost relevance of sensory properties in the experience of pleasure.

### 5.2. Collative Properties

Secondly, collative properties, including variety, novelty and surprise, have gained much attention within research. In particular, the roles of these properties in the pleasurable eating experience have been researched [40,41,42]. Collative properties imply a comparison (a collation) of incoming perceptual inputs with previous experiences, and an evaluation of similarities and differences between a stimulus’s different elements [43]. In a focus group study published by Andersen and Hyldig [37], the participants mentioned variation, novelty, positive surprise and familiarity to be important for the feeling of satisfaction. This finding clarifies that humans are consciously aware of the importance of these properties, but the research at the same time showed individual differences in the extent to which each collative property drove pleasure. According to Berlyne [44], the relationship between the hedonic appreciation of a stimulus and its arousal potential can be explained as a bell-shaped function, and individual optimum levels of arousal can be found. Contributing to the arousal potential are products’ collative properties [43]. Koster and Mojet [36] and Van Trijp and colleagues [45] studied the individual optimal arousal levels. Mojet and Koster found low optimal arousal levels among highly neophobic consumers—preferring stimuli they were familiar with—whereas consumers defined as variety seekers preferred more novel and complex stimuli. These findings stress the importance of not addressing a specific level of collative properties on a scale to measure food pleasure, but instead to focus on the pleasurable impact of the collative property per se (e.g., variety per se), as collative properties can be used to detect people with specific pleasure characteristics. Scales applied in clinical research to measure pleasure and anhedonia likewise acknowledge that collative properties can be used to detect anhedonic traits. The TEPS scale addresses variety by a focusing on “openness to different experiences” (the TEPS scale [15]) and “new foods” (the CPAS-scale [14]).

### 5.3. Expectations and Desires

Thirdly, according to Cardello and co-workers, perceptions of foods are guided by expectations developed during previous exposures and current available information [46,47,48]. Thus, expectations affect the ways in which consumers appreciate foods. Consumers are known to express some degree of pleasure with a stimulus that corresponds to their expected pleasure with that stimuli. This takes place mainly via assimilation processes, where perception of the stimuli is similar to the expectation. The “affective expectation model” posits that the degree of pleasure is formed based on a comparison to expectations of the stimuli, such that the expectation often determines the hedonic reaction [49]. As such, the more consumers expect to like, e.g., foods and drinks [50], the more they like them, once the food and drink are experienced. Consumers show a desire to preserve special memories, which in turn shows that memories provide utility and that nostalgia is hedonically reinforcing [51,52]. For some consumers, the desire to preserve memories is so strong that they not only try to procure memories, but also refrain from re-experiencing the event behind the memory, so as not to risk disturbing the pleasurable sensation connected to the memory [53]. Some consumers even show a desire to obtain novel and unusual experiences compared to more familiar and expectedly enjoyable experiences, in part so that they can enjoy recalling the experience later [54].

### 5.4. Post-Ingestive Sensations

Fourth, though the majority of studies investigating food-related pleasure measure and apply liking, wanting and other pleasure-associated tasks prior to or during consumption, pleasure from eating also depends on the sensations experienced post food intake. Several researchers have pointed to the fact that eating-related pleasure depends also on the mental and bodily wellbeing experiences after intake, and that this area of research deserves further study [55,56]. Studying post-ingestive drivers of pleasure requires measurements of interoceptive states [56,57], defined as the subjective experiences of internal signals related to, e.g., satiety, hunger, heat, pain, energy and visceral and muscular sensations [57,58,59,60]. Recently, Duerlund and colleagues demonstrated that mental wellbeing, overall wellbeing and physical wellbeing were highly influential to food-related pleasure [60]. Further, they showed inter-individual differences in the importance of appetite-related sensations (satiety, hunger, desire-to-eat) and feeling in need of food for pleasure, and in the vitality and energy-related post-ingestive variables (relaxation, energised and concentration). This research supports earlier research by Duerlund and colleagues [61], Sulmont-Rosse and colleagues [62], Andersen and colleagues [37,38,39] and Ares and colleagues [63], all supporting a link between post-ingestive sensations and pleasure.

### 5.5. Product Information

Fifth, access to product information [37,53]—explicitly, knowledge about geographical origins, has been found to add hedonic value to the eating experience in some people [64]. The pleasure generated from knowing about a food’s history has been studied previously. Stefani and colleagues showed that knowledge about food’s origins affected hedonic ratings [65]. Origins are hypothesised to affect a consumer’s evaluation in two ways: either as a quality cue by hinting to other characteristics, such as sensory characteristics, or by their symbolic role, i.e., ethical values, authenticity or the ability to awake memories of past experiences. Stefani and colleagues [65] found that information about origins acted as a quality cue. The more precisely the researchers defined the area the food originated from, the higher the quality expectations were. In line with this, access to product information was mentioned to be important for several participants in the focus group by Andersen and Hyldig [37]. In this study, it was found that product information could be a source of satisfaction by bringing knowledge about the food’s history, e.g., origin, production method and animal welfare, and healthiness, e.g., via information about ingredients. On a more general level, the importance of knowing about food’s history for pleasure can be related to the fact that this provides insights on the food’s production chain, and allows the consumers to make choices reflecting their personal values, e.g., values around organic production [50].

### 5.6. Eating Context

Finally, a group of key components previously found to influence food pleasure, which will be mentioned here, is related to the food context. Research suggests that at least two major concurrent context effects can alter the pleasure of the eating experience: the environment in which the food is eaten and the social interactions during consumption. Previous research has found that acceptance of the food can be very different depending on the location [66,67,68,69].

King and colleagues [67] studied the effects of meal situation, social interaction, physical environment and choice on food acceptability, and found that location had an effect on acceptability. From the focus group studies conducted by Andersen and Hyldig, participants explained that expectations were location-dependent, meaning that expectations of the meal varied depending on the location, which could alter the level of satisfaction [37]. Brown and colleagues [70] conducted a qualitative study focusing on the hedonic impression of the whole eating experience, including the social context. They found that participants thought of eating as a social act, and participants expressed that social eating could increase the overall hedonic eating experience and the feeling of satisfaction [70]. This finding was confirmed in the qualitative study by Andersen and Hyldig’s study [37]. They found that the social eating context for some participants was important for the feeling of satisfaction. Together, the research shows that both the physical and the social context can alter the hedonic experience.

The food context is addressed in several existing scales for measuring anhedonia. The TEPS scale addresses the physical context directly via a focus on satisfaction from drinking a hot cup of coffee or tea on a “cold morning” and eating “out in a restaurant” [15]. The DARS [11], SHAPS [12], CPAS [14] and FCPS [13] all focus on the hedonic experience of social interaction, but not in a food context.

## 6. A Conceptual Framework of Food Pleasure

Based on the current scales measuring (an)hedonia and the broad range of studies focusing on the pleasurable aspects of food-related experiences, our main hypothesis is that several dimensions contribute to the complete pleasurable response to food-related experiences. A deficiency in any of these dimensions could theoretically reduce the experience of eating related pleasure, and the importance of each dimension is subject to inter-individual differences. One concrete example of a dimension contributing to overall food pleasure is “the sensory food experience,” and another dimension is “collative properties.” Every one dimension is part of the total pleasure experience; however, the dimensions can be studied individually. The level of pleasure generated from any one dimension differs between subjects (potentially consumer segments), and a subject can consequently suffer from dimension-specific anhedonia.

A second hypothesis is that a pool of items contribute to each dimension, and the importance of each item is subject to inter-individual differences, which can affect the dimension-related pleasure. For the dimension related to “the sensory food experience,” these items could naturally include: the food’s appearance, odour, texture and taste. Several studies have previously shown subjective differences in the importance of sensory properties for overall liking and satisfaction [71,72,73], and for food pleasure, when suffering from sensory impairments [74,75].

A third hypothesis is that different behaviours are involved in the pleasure related to each item, and these behaviours are distinct in nature; e.g., the disposition to experience anticipatory pleasure to food taste, to exemplify one item, can be distinguished from the disposition to experience consummatory pleasure of the food taste. Based on the review of existing scales to measure anhedonia in clinical settings (not food-focused scales), different behaviours have been found to be involved in pleasure related to each dimension. Commonly, “anticipatory” and “consummatory” pleasures have been identified as key behaviours in the experience of pleasure. Anticipatory pleasure is distinguished from consummatory pleasure in that anticipatory pleasure refers to the pleasured experienced in anticipation, which is closely related to motivation and goal-directed behaviour [76]. Consummatory pleasure refers to an experience-based enjoyment and is closely linked to fulfilment of a desire. In the context of food pleasure, these two behaviours have predominately been referred to as “wanting” and “liking,” respectively, with “wanting” referring to motivation to engage in food-related experiences (e.g., intake) and “liking” referring to the pleasure derived from being subjected to the food-related experience (e.g., eating) [77,78,79].

Inclusion of these behaviours in the Food Pleasure Scale is expected to capture a comprehensive representation of the individual’s pleasurable eating experience. Further, a questionnaire tapping into various dimensions involved in food-related pleasure and the behaviours related to each dimension will have the potential to elucidate whether food-related an-hedonia is dimension specific, item specific, behaviour specific or a combination. Regarded as such, the pleasurable response to food-related experiences is believed to be multi-layered, and individuals can experience varying degrees of pleasure depending on the dimension, item and/or behaviour in focus. A conceptualisation of the Food Pleasure response is presented in Figure 1.

As a questionnaire is based on reflection (when presented in the absence of food consumption), a questionnaire method is believed to be optimal for detecting people with anhedonia or anhedonic traits. This is due to the commonly occurring hypothesis that anhedonia does not affect the subjective feeling of pleasure during rewards themselves [80]. Instead, it is hypothesised to affect the way we think about past or future pleasure. E.g., people with anhedonia like chocolate as much as anybody else, but when they are not eating it, they think they like it less. Put in another way, people with anhedonia seem to experience consummatory pleasure similar to other people, but they are less likely to anticipate and remember everyday pleasures, and are less willing to pursue them, especially if that requires a lot of effort or investment. The hypothesis by Soukupova [80] confirms the finding of Berridge [77], Gard, Gard, Kring and John [15] (2006) and Rizvi et al. [11]: that such a questionnaire should address anticipation and consummatory pleasure (experienced), as a low rating in any of these behaviours (despite the dimension in focus) would indicate food anhedonia.

## 7. Conclusions

Various factors have been found to influence the hedonic eating response and thereby to influence eating behaviour. Each factor can be regarded a piece that contributes to parts of the total picture of the hedonic response to food. As a result, the literature on the hedonic response to food-related experiences is comprehensive, but at the same time rather fragmented; and importantly, it is not clear how individuals/segments differ in key drivers of their hedonic experiences and the extents to which food pleasure is perceived.

In this paper, we presented a conceptual framework for the development of a scale (self-report questionnaire) to measure qualitative and quantitative aspects of food-related pleasure, the Food Pleasure Scale. We introduced the concept of (an)hedonia and scales developed in the past for its measurement, and identified the spectrum of dimensions influencing food-related pleasure—sensory and collative properties, expectations and desires, post-ingestive sensations and product and context related factors. We identified items and behaviours linked to each dimension.

We presented the Food Pleasure Scale as the next step in research aiming to clarify individual differences in quantitative and qualitative aspects of pleasure from food-related experiences. This scale will facilitate researchers accurately tapping into the subjective nature of what individuals find pleasurable in food-related contexts; identify subjects with altered or impaired hedonic responses and the characteristics of these populations; and clarify the underlying causes when combined with other means, such as neuroscience and methods addressing the subconscious aspects of reward. Greater insights into food pleasure cues will be important for understanding the unique flexibility of human food choices and the conditions that might promote eating behaviours (including overeating), and be relevant for understanding and guiding people toward healthy eating behaviour.

## Figures and Tables

**Table 1:** Overview of key food and eating-related behavioural factors influencing food-related pleasure.

Component Influencing Food-Related Pleasure	Item in Focus	Hedonic Variable	Reference
Intrinsic product characteristics	Sensory properties	Acceptance	Harper, 1981 [29]; Land, 1983 [30]; Tuorila, 2007 [31];
		Preference	Khan, 1981 [32];
		Eating Behaviour	Cardello, Bell, & Kramer, 1996 [33]; Connors, Bisogni, Sobal, & Devine, 2001 [34]; Fürst et al., 1996 [35]; Koster & Mojet, 2007 [36];
		Satisfaction	Andersen & Hyldig, 2015a [37]; 2015b [38]; Andersen, Mielby, Viemose, Bredie & Hyldig, 2017 [39];
	Collative properties	Eating Behaviour	Berlyne, 1950 [40]; 1966 [43]; 1970 [44]; Koster & Mojet, 2007 [36]; Van Trijp, Lahteenmaki & Tuorila, 1992 [45];
		Preference	Mielby, Kildegaard, Gabrielsen, Edelenbos, & Thybo, 2012 [41]; Giacalone, Duerlund, Bøegh-Petersen, Bredie & Frøst, 2014 [42];
		Satisfaction	Andersen & Hyldig 2015a [37]; Andersen, Mielby, Viemose, Bredie & Hyldig, 2017 [39];
Expectation and desires	Expectations	Liking	Lee, Frederick & Ariely, 2006 [50];
		Acceptance	Cardello et al., 1985 [46]; Cardello & Sawyer, 1992 [47]; Cardello, 1994 [48];
		Affective reaction in general	Wilson & Klaaren, 1992 [49];
	Nostalgia	Preference	Loveland, Smeeters & Mandel, 2008 [51]; Wildshut, Sedikides, Arndt & Routledge, 2006 [52];
	Memory	Eating Behaviour	Alba & Williams, 2013 [53];
Post-ingestive sensations	Physical and mental sensation	Food-related pleasure	Duerlund, Andersen, Wang, Chan & Byrne, 2020 [60]; Duerlund, Andersen, Grønbeck & Byrne, 2019 [61];
		Satisfaction	Andersen & Hyldig, 2015a [37]; 2015b [38]; Andersen, Mielby, Viemose, Bredie & Hyldig, 2017 [39];
		Wellbeing	Ares et al., 2015 [63];
		Feeling good	Sulmont-Rossé et al., 2019 [62];
Product information	Access to product information in general	Eating behaviour	Alba & Williams, 2013 [53];
		Satisfaction	Andersen & Hyldig, 2015a [37];
	Location of origin	Liking	Verlegh & Van Ittersum, 2001 [64]; Stefani, Romano, & Cavicchi, 2006 [65]; Andersen & Hyldig 2015a [37];
	Production method	Satisfaction	Andersen & Hyldig 2015a [37];
		Eating Behaviour	Lee, Shimizu, Kniffin & Wansink, 2013 [50]
Eating context	Physical setting	Acceptance	de Graaf et al., 2005 [66]; King et al., 2004 [67]; Meiselman, Johnson, Reeve & Crouch, 2000a [68]; Edwards & Meiselman, 2005 [69];
		Satisfaction	Andersen & Hyldig, 2015a [37];
	Social setting	Acceptance	King et al., 2004 [67];
		Emotions	Brown, Edwards & Hartwell, 2013 [70];
		Satisfaction	Andersen & Hyldig, 2015a [37];

## Data Availability

The datasets generated for this study are available on request to the corresponding author.
